# Comparative transcriptome analysis reveals putative transcription factors components of the regulatory network for cannabidiol biosynthesis in *Cannabis sativa* L

**DOI:** 10.3389/fpls.2026.1914974

**Published:** 2026-08-31

**Authors:** Ying Li, Huijuan Tang, Tian Zhang, Xiaolin Li, Bowen Zheng, Meng Cheng, Guang Yang, Gen Pan

**Affiliations:** 1 National Resource Center for Chinese Materia Medica, China Academy of Chinese Medical Sciences, State Key Laboratory Breeding Base of Dao-di Herbs, Beijing, China; 2 Institute of Bast Fiber Crops, Chinese Academy of Agricultural Sciences, Changsha, China; 3 Institute of Chinese Medicine Resources, Hunan Academy of Chinese Medicine, Changsha, China

**Keywords:** cannabidiol, Cannabis, comparative transcriptome, regulate, transcription factor

## Abstract

**Background:**

Cannabidiol (CBD), a unique secondary metabolite of *Cannabis sativa* L. (cannabis), is widely used in medicine, food, cosmetics, and forage. Elucidation of the transcriptional regulation mechanism can provide a theoretical basis for molecular breeding with higher CBD content. However, the regulatory network for cannabidiol biosynthesis in cannabis is largely unknown.

**Methods:**

Transcriptome analysis of four cannabis tissues was conducted to screen differentially expressed genes. Short time-series expression miner (STEM) analysis combined with strain-specific expression correlation analysis was performed to filter core transcription factors (TFs) associated with CBD biosynthetic enzyme genes. Yeast one-hybrid and yeast two-hybrid assays were applied to verify the binding and protein interaction relationships of key TFs, and subcellular localization assay was performed in tobacco leaf.

**Results:**

A total of 1314 genes specifically upregulated in female flowers were identified by transcriptome analysis of four different tissues with distinct CBD contents and clustered into 19 profiles using short time-series expression miner (STEM) analysis. Profile 8, including 16 TFs and 6 enzyme genes related to the CBD biosynthesis pathway, demonstrated similar trends in the CBD contents of the four tissues. Among the 16 TFs, 7 exhibited similar strain-specific expression profiles and their expression levels positively correlated with those of the six enzyme genes. Moreover, a yeast one-hybrid assay found that CsMYB6 and CsMYB29 bind to the promoters of four and two CBD biosynthetic enzyme genes, respectively. Meanwhile, a yeast two-hybrid assay revealed that CsMYB6 interacts with two regulators of CBD biosynthesis, CsMYB1 and CsWRKY1, while CsMYB29 interacts with CsWRKY1. In addition, CsMYB29 and CsMYB6 were localized in the nuclei.

**Conclusions:**

In summary, the two TFs, CsMYB6 and CsMYB29, identified in this study provide valuable insight into the complex regulatory mechanisms of CBD production in cannabis and offer a genetic resource for the genetic improvement of CBD content.

## 1 Introduction

*Cannabis sativa* L. (Cannabis) is an important economic crop and a diploid annual herbaceous plant cultivated worldwide (McPartland and Small, 2020). Historically, cannabis has been cultivated as an important food, fiber, and medicinal plant with application in textiles, food, building materials, medicines, and other field. Cannabis plants reportedly contain more than 500 chemical compounds, including 125 cannabinoids (Khalsa et al., 2022). Among these cannabinoids, cannabidiol (CBD) and tetrahydrocannabinol (THC) are two common types with numerous physiologically active functions. THC, a psychoactive cannabinoid with addictive properties, is banned in several countries. By contrast, CBD is a non-psychoactive cannabinoid that has been shown to have anti-inflammatory and pain relief properties and is currently widely used in many products, including foods, pharmaceuticals, health products, drinks, and cosmetics (Salami et al., 2020). Therefore, cannabis cultivation for CBD production has become increasingly important with increasing medical and economic demand.

Increasing the CBD content in cannabis plants is the main goal of cannabis variety development, particularly in China, where there is a severe shortage of high-CBD varieties (Zhang et al., 2019). However, cannabis is largely dioecious and heterogamous, with cross-pollinated leading to high natural hybridization rates and heterozygosity, which results in traditional breeding having the disadvantages of long breeding periods and low efficiency (Zhang et al., 2019). Research on the biosynthetic and regulatory molecular mechanisms of CBD can provide a theoretical basis for the targeted enhancement of CBD content. To date, CBD biosynthesis pathways has been identified and include three main biosynthetic pathways (**Supplementary Figure 1**) (Hurgobin et al., 2021; Xie et al., 2023). In the polyketide synthesis pathway, the polyketide nucleation component, olivetolic acid (OA), is formed in the cytosol from hexanoic acid via three enzymes: acyl-activating enzymes (AAE), olivetolic acid cyclase (OAC) enzymes, and olivetol synthase (OLS). The second pathway is the methylerythritol 4-phosphate (MEP) pathway, which occurs in the plastid matrix and produces geranyl pyrophosphate (GPP) through enzymes such as geranyl pyrophosphate synthase (GPPS). The final pathway is cannabinoid synthesis, in which OA and GPP are catalyzed into cannabigerolic acid (CBGA) by cannabigerolic acid synthase (CBGAS) in the plastid membrane. Cannabidiolic acid (CBDA) is then synthesized from CBGA by CBDA synthase (CBDAS). CBDA is further converted into CBD via non-enzymatic decarboxylation (Van et al., 2011; Xie et al., 2023).

Previous studies have confirmed that CBDAS is a key enzyme gene in CBD biosynthesis (Onofri et al., 2015; Zirpel et al., 2018). However, despite a 10-fold upregulation of *CsCBDAS* gene expression in overexpressing plants compared with wild-type plants, the CBD content of the plants only increased by 54% (Desgagné-Penix and Facchini, 2012). In addition, more than one locus that controls CBD content has been identified (Laverty et al., 2019; Grassa et al., 2021). These studies suggest that manipulation of the *CBDAS* gene alone is insufficient for the development of high-CBD varieties. Therefore, an in-depth analysis of the transcriptional regulation mechanism of CBD synthesis is required to provide genetic resources and a theoretical basis for the targeted cultivation of high-CBD varieties. To date, some regulatory factors involved in this process have been identified in cannabis. Using a yeast one-hybrid (Y1H) assay, it was demonstrated that two transcription factors, CsMYB1 and CsWRKY1, activate gene expression through the *CBDAS* promoter (Liu et al., 2021). Another transcription factor, CsMYB106, which is induced by cadmium stress, was also found to regulate cadmium-mediated CBD biosynthesis and the development of glandular trichome, which are responsible for CBD production (Yin et al., 2022; Haiden et al., 2022). Despite these findings, the number of transcription factors known to be associated with CBD biosynthesis is limited compared to the complexity of the regulatory network, and the underlying mechanisms remain largely unknown.

In this study, four tissues with distinct CBD contents were used for transcriptome analysis, and tissue-specific transcription factors (TFs) were identified using bioinformatics. Putative key TFs were further investigated through expression profiling in 5 cannabis varieties, short time-series expression miner (STEM), correlation, and subcellular localization analyses. The regulatory relationships between TFs and *CsCBDAS* were verified using yeast one-hybrid (Y1H) and yeast two-hybrid (Y2H) assays. Thus, our results provide a biological basis for elucidating regulatory networks for CBD biosynthesis and genetic information for developing high-CBD varieties via molecular breeding methods.

## 2 Materials and methods

### 2.1 Cannabis varieties and growth conditions

Plants of the ‘C4,’ ‘C7, ‘C2’, ‘YDM1,’ and ‘YM6’ varieties were obtained from the Institute of Bast Fiber Crops at the Chinese Academy of Agricultural Sciences, and grown in a greenhouse under conditions described previously (Jiang et al., 2022). The growth conditions in the greenhouse were as follows: long-day treatment (18 h light/6 h dark cycle for 24 h, 26 ± 2°C), and when the plants entered reproductive growth, short-day treatment were performed (8 h light/16 h dark cycle for 24 h, 26 ± 2°C). The four tissues of ‘C4’ female plants at flowering stage, including the upper female flower, root, upper stem, and upper leaves, were further used for transcriptome sequencing.

### 2.2 RNA extraction, transcriptome sequencing, and analyses

Total RNA from different tissues of ‘C4’ (female flower, root, leaf, and stem) and the female flower of different varieties (‘C7’, ‘C2’, ‘YDM1’, and ‘YM6’) were extracted with the EASYspin Plus Plant RNA Kit (Aidlab Biotechnologies Co., Ltd., Beijing, China) according to previously published protocols (Zhu et al., 2020). A total of 1 µg RNA from the four tissues of the ‘C4’ variety was further reverse transcribed with NEBNext^®^ Ultra™ II RNA Library Prep Kit (New England Biolabs Inc., Ipswich, MA, USA) as described previously and then used for RNA sequencing performed by Shanghai OE Biotech Co., Ltd. (Shanghai, China) (Zhou et al., 2022).

The raw data were processed and filtered using Trimmomatic to remove poly-N and low-quality reads. The fragments per kilobase of the exon model per million mapped fragments (FPKM) value for each unigene was calculated using Cufflinks. Hisat2 was used to align clean reads with the reference genome and calculate the mapping rate, and the reference genome was obtained from the NCBI Biotechnology Information database (https://www.ncbi.nlm.nih.gov/) (GCA_900626175.2). DESeq2 was used to identify differentially expressed genes (DEGs) in the comparing groups (Female flower versus Root, Female flower versus Leaf, Female flower versus Stem) with the following parameters: *P* < 0.05 and fold change (FC) > 2 or < 0.5. Genes with FC > 2 indicate significant upregulation in Female flower relative to Root or Leaf or Stem, while genes with FC < 0.5 indicate significant downregulation in Female flower. The read counts were normalized to the FPKM value to represent the gene expression level. Gene Ontology (GO) and Kyoto Encyclopedia of Genes and Genomes (KEGG) pathway enrichment analyses were performed using all DEGs (He et al., 2020). STEM analysis was performed as previously described (Ernst and Bar-Joseph, 2006).

### 2.3 Gene regulatory network analysis

The FPKM values of 16 transcription factors (TFs) and six enzyme genes from profile 8 of the transcriptome data across four tissues were used to construct the gene regulatory network (GRN) with the software GENIE3 (v1.12.0) (https://bioconductor.riken.jp/packages/release/bioc/vignettes/GENIE3/inst/doc/GENIE3.html). A threshold (weight≥0.2) was applied to obtain a regulatory network diagram highlighting strong regulatory relationships between 13 TFs and 6 enzyme genes. The GRNs were visualized using Cytoscape (v3.7.2).

### 2.4 Determination of total CBD and THC content

Four tissues (female flower, root, leaf, and stem) were harvested from flowering-stage’C4’ cannabis plants, while only female flowers were collected from flowering’C7’, ‘C2’,’YDM1’, and ‘YM6’ genotypes. All plant materials were subjected to cannabinoid extraction, and CBD quantification was performed using reversed-phase high-performance liquid chromatography coupled with ultraviolet detection (RP-HPLC-UV), following the analytical procedure established by Mandrioli et al. (2019). Tissues were dried at 35 ± 1°Cto constant weight, subsequently ground, and then sieved through a 1 mm mesh, and the resulting powder was stored at −20°Cunder nitrogen until further extraction. Approximately 25 mg of powder was subjected to a two-step extraction procedure with 10 mL methanol-chloroform (9:1, v/v), with each extraction consisted of oscillation (10 min, 350 rpm) followed by ultrasonication (10 min), after which the mixture was centrifuged (10 min, 1,125×g). The combined supernatants were then brought to 25 mL, filtered through a 0.45 μm membrance, and subsequently dried under a stream nitrogen and the residue was finally reconstituted in 500 μL acetonitrile. For quantitative analysis, calibration curves for CBDA, CBD and Δ^9^-THC (THC) were constructed at eight concentrations ranging from 0.05 to 100 μg/mL. RP-HPLC-UV analysis was performed under the following conditions: column temperature 35°C,autosampler temperature 4°C, UV detection at 220 nm, injection volume 5.0 μL, and flow rate 1.6 mL/min. Gradient elution was carried out as follows: 70% B (0–3 min), 85% B (3–7 min), 95% B (7.01-8.00 min), and 70% B (8.01–10 min), where eluent A consisted of water containing 0.085% phosphoric acid and eluent B consisted of acetonitrile containing 0.085% phosphoric acid. Total CBD were calculated as CBD + (0.877× CBDA), and all analyses were performed in three independent replicates.

### 2.5 Subcellular localization of CsMYB6 and CsMYB29 in tobacco leaf cells

The method for subcellular localization was described previously (Pan et al., 2024). In brief, cDNA templates of the ‘C4’ leaf were used to amplify the CDS of *CsMYB29* and *CsMYB6*. Subsequently, the target sequences of *CsMYB29* and *CsMYB6* were obtained through PCR amplification and the successful amplification of products of the expected size was verified by gel electrophoresis. Then, the target sequences were recovered and ligated to the linearized pBI121-GFP vector. The recombinant plasmid was then transferred in T1 cell, and the positive clones were further confirmed by PCR. The recombinant plasmids were further transformed into GV3101 cells, and the positive clones were grown in LB medium until OD_600_ reached 0.6. Then, these cells were pelleted by centrifugation (5000 rpm, 10 min), and resuspended twice in MS liquid medium. After being left at room temperature for 3 h, the tobacco leaves injected with a 1 mL needle were observed after 48 h using laser scanning confocal microscopy (LSM880, Zeiss).

### 2.6 qRT–PCR analysis

The female flowers of ‘C7,’ ‘C2,’ ‘YDM1,’ and ‘YM6’ were used for qRT-PCR analysis, and each sample included 3 biological replicates. Reverse transcription was performed using the Evo M-MLV RT Premix for qPCR Kit (Accurate, Changsha, China) according to the manufacturer’s instructions. qRT–PCR reactions were performed on a CFX Connect real-time system (Bio-Rad) using the SYBR^®^ Green Premix Pro Taq HS qPCR Kit according to published protocols (Bao et al., 2016). The *EF1a* gene was used as an internal control (Liu et al., 2021). The primers used for qRT–PCR analysis are listed in **Supplementary Table 1**. Relative expression levels were calculated using the 2^−∆∆Ct^ method.

### 2.7 Y1H assay

For the Y1H assay, approximately 1 kb promoter regions of five enzyme genes and the full-length CDS of the TFs were amplified using the DNA of the ‘C4’ female flower and detected by gel electrophoresis. Then, the target sequences were recovered and ligated to the linearized pLacZi and pB42AD vectors, respectively (Lin et al., 2007). After PCR amplification and detection by gel electrophoresis, the positive recombinant plasmids, including pLacZi and pB42AD, were co-transformed into EGY48 and then grown in SD/-Leu/-Trp mediam at 30°Cfor 3–5 days. The clones were further grown in SD/-Leu/-Trp/-His/-Ade/X-α-gal for 2–3 days at 30°C. DNA-protein interactions were reflected by the ability of the co-transformants to grow on SD/-Leu medium containing Aureobasidin A. Primers used for the Y1H assay are listed in **Supplementary Table 1**.

### 2.8 Y2H assay

The CDS of *CsMYB1*, *CsMYB6*, *CsMYB29* and *CsWRKY1* were amplified from the cDNA of ‘C4’ using the primers listed in **Supplementary Table 1**. *CsMYB6* and *CsMYB29* were obtained through PCR amplification and detected by gel electrophoresis. Then, the target sequences of *CsMYB6* and *CsMYB29* were recovered and ligated to the linearized pGADT7, while the target sequences of *CsMYB1* and *CsWRKY1* were recovered and ligated to the linearized vector pGBKT7. After construction, both positive vectors were co-transformed into the yeast strain AH109 and grown on DDO (SD-Leu/-Trp) plates for 3 days at 30°C. Protein–protein interactions were then further investigated on QDO (SD-Leu/-Trp/-His/-Ade/X-α-gal) media incubated for 3–5 days at 30°C. After PCR amplification and detection by gel electrophoresis, the recombinant plasmid including pGADT7 and pGBKT7 were transferred into AH109 and then grown in SD/-Leu/-Trp mediam at 30°Cfor 3–5 days. The clones were further grown in SD/-Trp/-Ura/Gal/Raf/X-Gal.

## 3 Results

### 3.1 Determination of CBD content in different tissues

**Figure 1:**
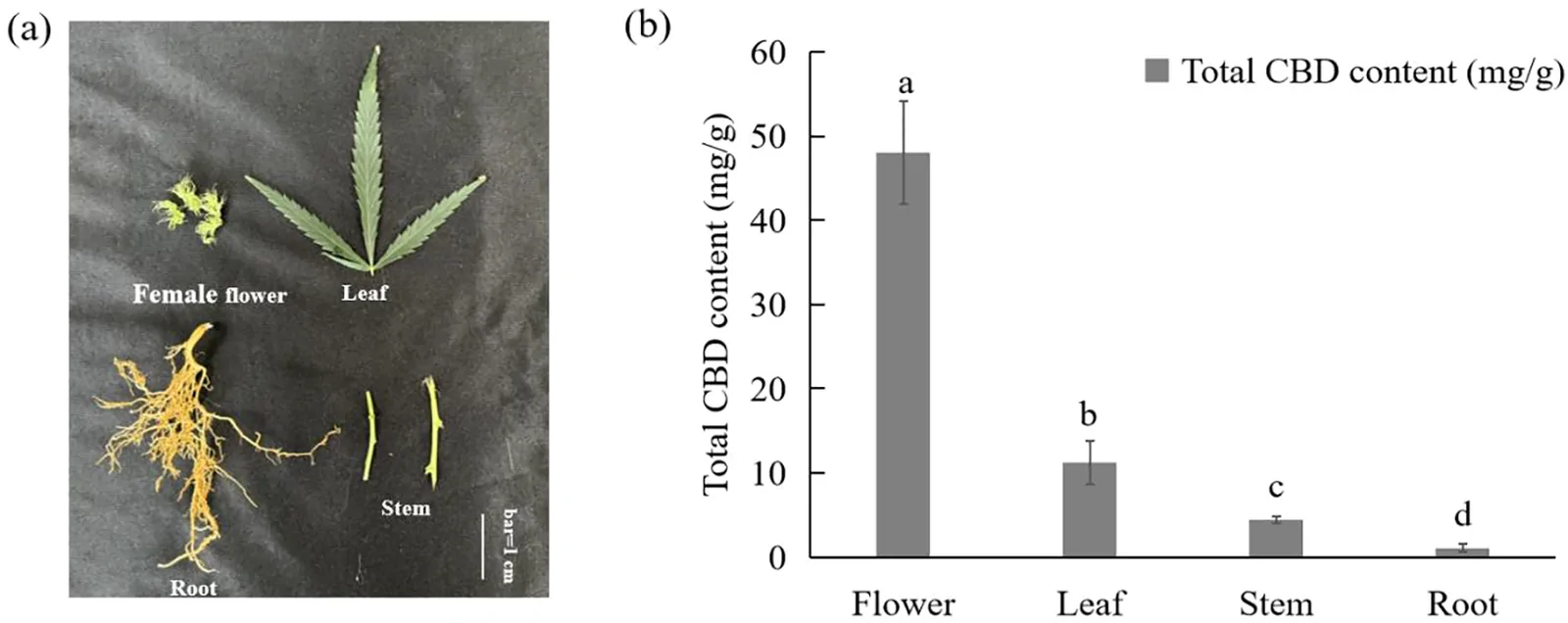
Phenotype of four different tissues and its cannabidiol (CBD) content measurement **(a)** Phenotype of upper female flower, upper leaf, root and upper stem of ‘C4’ variety plants at flowering stage; **(b)** Determination of total CBD content of different tissues. Different letters indicate significant difference between different tissues (*P* < 0.05). Data are means (± SD), and each experiment included three biological replicates. Scale bars: 1 cm.

The total CBD contents of the four tissues from female plants were determined. As shown in **Figure 1**, the total CBD contents differed significantly among the four tissues: the female flower contained the highest total CBD content (48.03 mg/g), followed by the leaf, stem, and root (1.09 mg/g) (**Figure 1a**). In addition, the content of THC was also highest in female flower compared with the other three tissues (**Supplementary Figure 2**).

### 3.2 Sequencing and assembly of cannabis transcriptome and identification of DEGs

**Figure 2:**
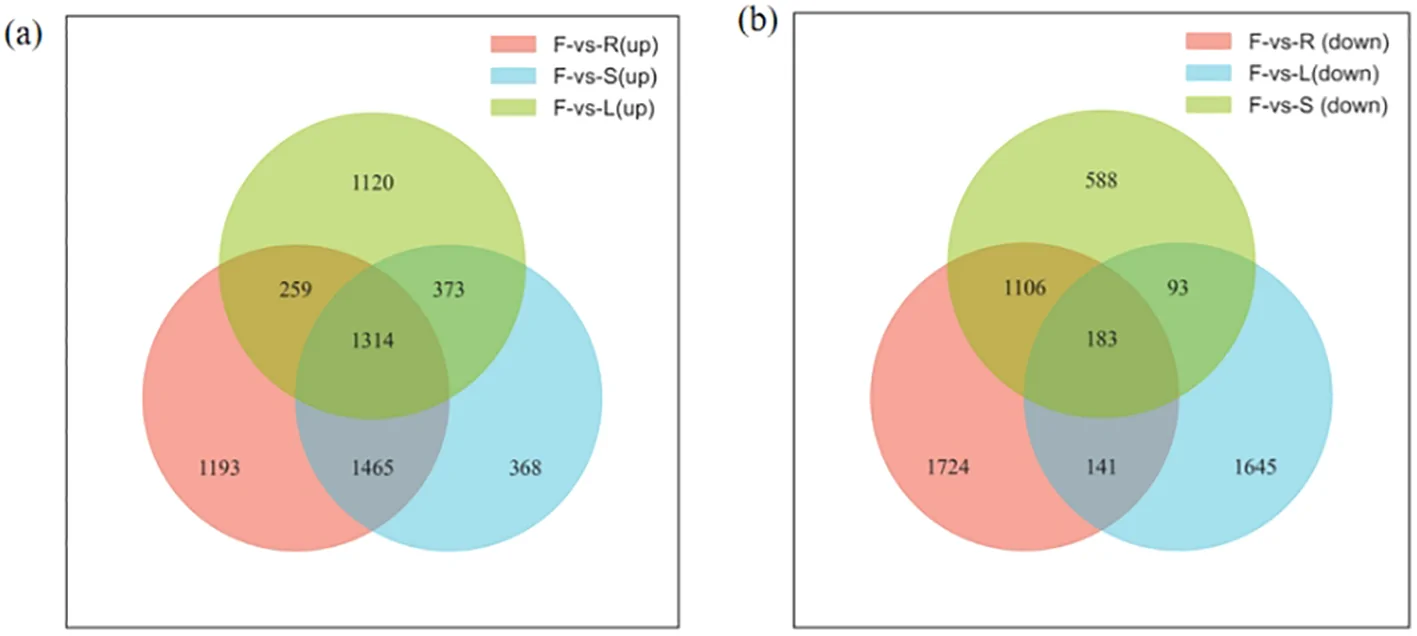
Venn diagram of common differentially expressed genes (DEGs) in Flower-vs-Root/Stem/Leaf groups. **(a)** Venn diagram of common up-regulated DEGs in female flower-vs-root/stem/leaf groups; **(b)** Venn diagram of common down-regulated DEGs in female flower-vs-root/stem/leaf groups. R, root; F, female flower; S, stem; L, leaf.

RNA-Seq was performed on four tissues including female flowers, roots, stems, and leaves from ‘C4’ female plants, with each tissue including three biological replicates. A total of 88.25 Gb of reads were obtained, with an average Q30 score of 94.77%, an average GC content of 0.4377, an average of 50.11M clean reads, and 7.35G of clean bases. The mapping rate ranged from 89.85% to 92.91%, with an average mapping rate of 92.24% (**Supplementary Table 2**). Across pairwise comparisons, female flowers contained 10,817 upregulated DEGs, including 3,066 (female flower vs. leaf), 3,520 (female flower vs. stem), and 4,231 (female flower vs. root). A total of 7,186 DEGs were downregulated, comprising 2,062 (female flower vs. leaf), 1,970 (female flower vs. stem), and 3,154 (female flower vs. root). After eliminating overlapping DEGs shared among the three comparisons, a total of 11,572 non-redundant DEGs were identified in female flowers relative to roots, stems, and leaves, of which 6,092 were upregulated and 5,480 were downregulated (**Figure 2**).

### 3.3 Female flower specific up-regulated DEGs

**Figure 3:**
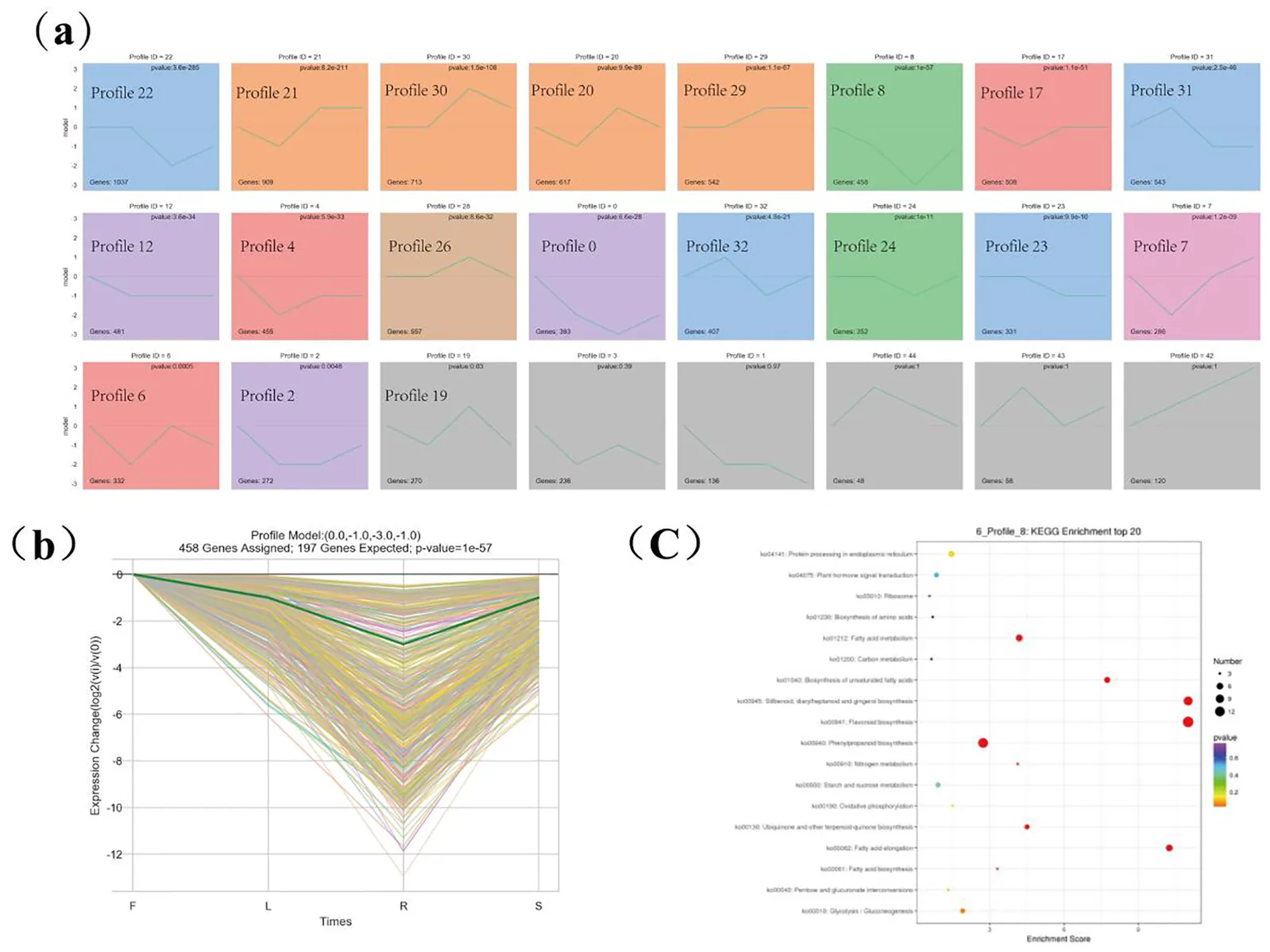
Short time-series expression miner (STEM) analysis of differentially expressed genes (DEGs). **(a)** 19 profiles analyzed by STEM analysis; **(b)** The profiles similar with the trend of the CBD content of four tissues; **(c)** The Kyoto Encyclopedia of Genes and Genomes (KEGG) pathway enrichment analyses of DEGs from the profile 8.

Female flowers contained the highest CBD contents among the tissue types. Therefore, female flower- specific up-regulated DEGs were further studied to identify key putative DEGs involved in the regulation of CBD biosynthesis. In the three pairwise comparisons, the Venn diagram of DEGs showed that 1,314 common DEGs were upregulated and 183 common DEGs were downregulated (**Figure 2**). DEGs in female flowers with similar expression trends were further analyzed using STEM. In total, there were 19 profiles for all DEGs, among which profile 8 demonstrated a trend similar to that of CBD content in the four tissues (**Figure 3**). Furthermore, profile 8 contained 197 genes, among which six encode biosynthetic enzymes involved in CBD biosynthesis, such as *AAE*, *CBDAS*, and polyketide synthase (PKSG), and 16 genes belong to transcription factors (TFs), including MYB and bHLH (**Table 1**). KEGG enrichment analysis showed that most of the DEGs from profile 8 were significantly enriched in ‘flavonoid’ and ‘phenylpropanoid biosynthesis’ (**Figure 3c**).

**Table 1:** Summary of DEGs in profile 8.

Types	id	Gene_symbol	Description
Enzyme genes	LOC115695955	*AAE6-1*	Probable acyl-activating enzyme 6
	LOC115697762	*CBDAS*	Cannabidiolic acid synthase
	LOC115699293	*PKSG4-1*	Polyketide synthase 4
	LOC115700696	*PKSG4-2*	Polyketide synthase 1
	LOC115725729	*AAE6-2*	Probable acyl-activating enzyme 6
	LOC115723437	*OAC*	Olivetolic acid cyclase
Up-regulated	LOC115696941	*ASR3*	Trihelix transcription factor ASR3
	LOC115698585	*MYB30*	Myb-related protein 30
	LOC115701410	*MYB106*	Transcription factor MYB106
	LOC115702403	*BHLH113*	Transcription factor bHLH113
	LOC115703590	*BHLH42*	transcription factor BHLH42
	LOC115704832	*ERF023*	Ethylene-responsive transcription factor ERF023
	LOC115705338	*WIN1*	Ethylene-responsive transcription factor WIN1
	LOC115706528	*MYB17*	Transcription factor MYB41
	LOC115711687	*MYB29*	Transcription factor MYB29
	LOC115712163	*MYB6*	Transcription repressor MYB6
	LOC115712532	*VRN*	B3 domain-containing transcription factor VRN1
	LOC115716337	*MADS6*	MADS-box transcription factor 6
	LOC115721889	*MYB4*	Transcription repressor MYB4
	LOC115725286	*MYB5*	Transcription repressor MYB5
	LOC115725520	*BHLH146*	Transcription factor bHLH146
	LOC115708734	*MYB122*	Transcription factor MYB122

**Figure 4:**
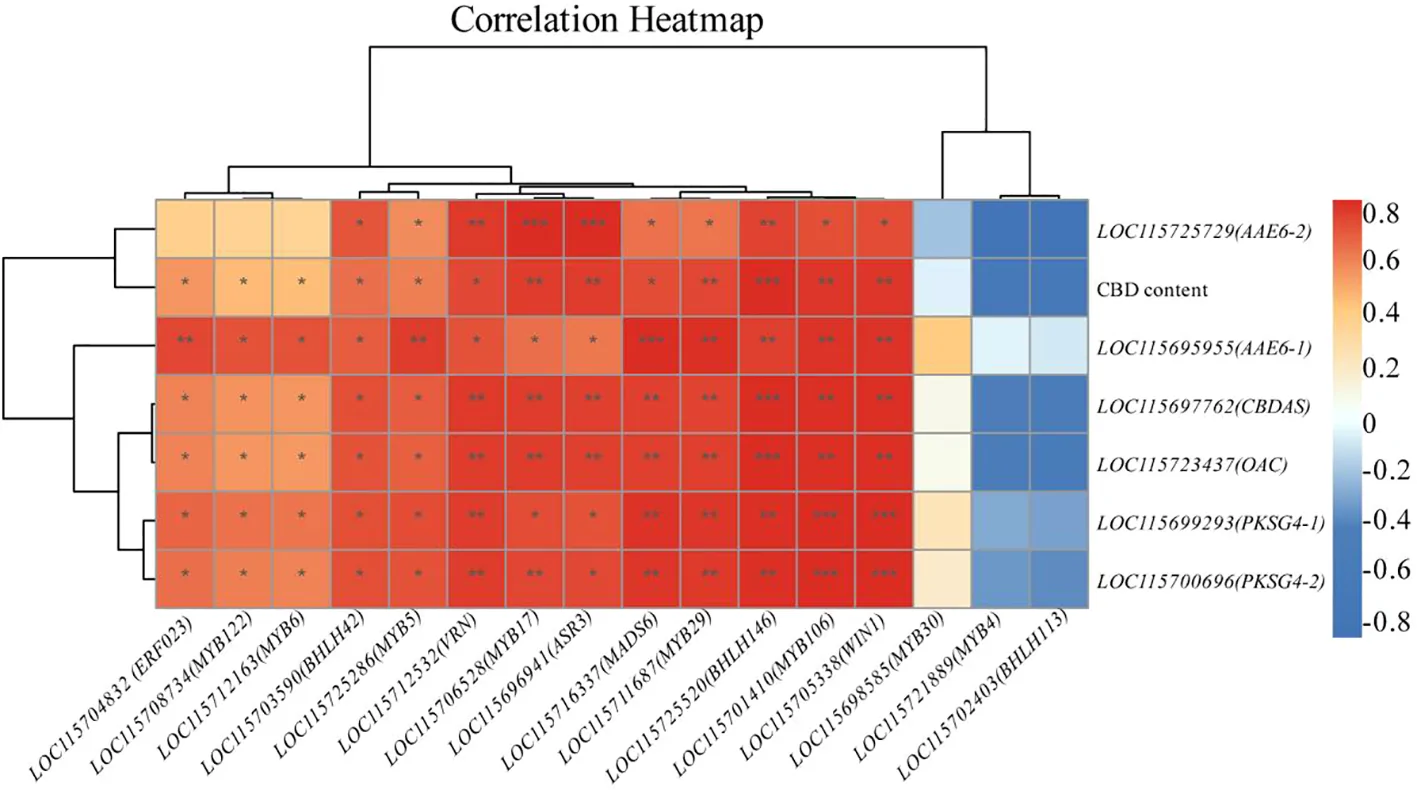
Correlation heatmap showing the relationship between transcription factor (TF) expression, enzyme gene expression, and cannabidiol (CBD) content across four tissues of cannabis cultivar ‘C4’. Expression data were derived from RNA-seq. Significant differences among groups were assessed by one-way ANOVA (P < 0.05), with different letters denoting statistically distinct groups. Correlation significance levels: *P* < 0.05. *P < 0.05; **P < 0.01; ***P < 0.0001.

To gain further insight into the regulatory mechanism of CBD biosynthesis, the correlations between the transcript levels of these 16 TFs and the six enzyme genes from profile 8 were analyzed. We found that the transcript levels of 10 TFs were positively correlated with the expression levels of the six enzyme genes, and the expression levels of 3 TFs showed a significant positive correlation with those of the five enzyme genes, except for *AAE6-2* (LOC115725729) (**Figure 4**). In addition, the expression levels of 13 upregulated TFs were positively correlated with CBD content (**Figure 4**; **Supplementary Figure 4**).

### 3.4 Expression profiling of TFs in varieties with different CBD levels

**Figure 5:**
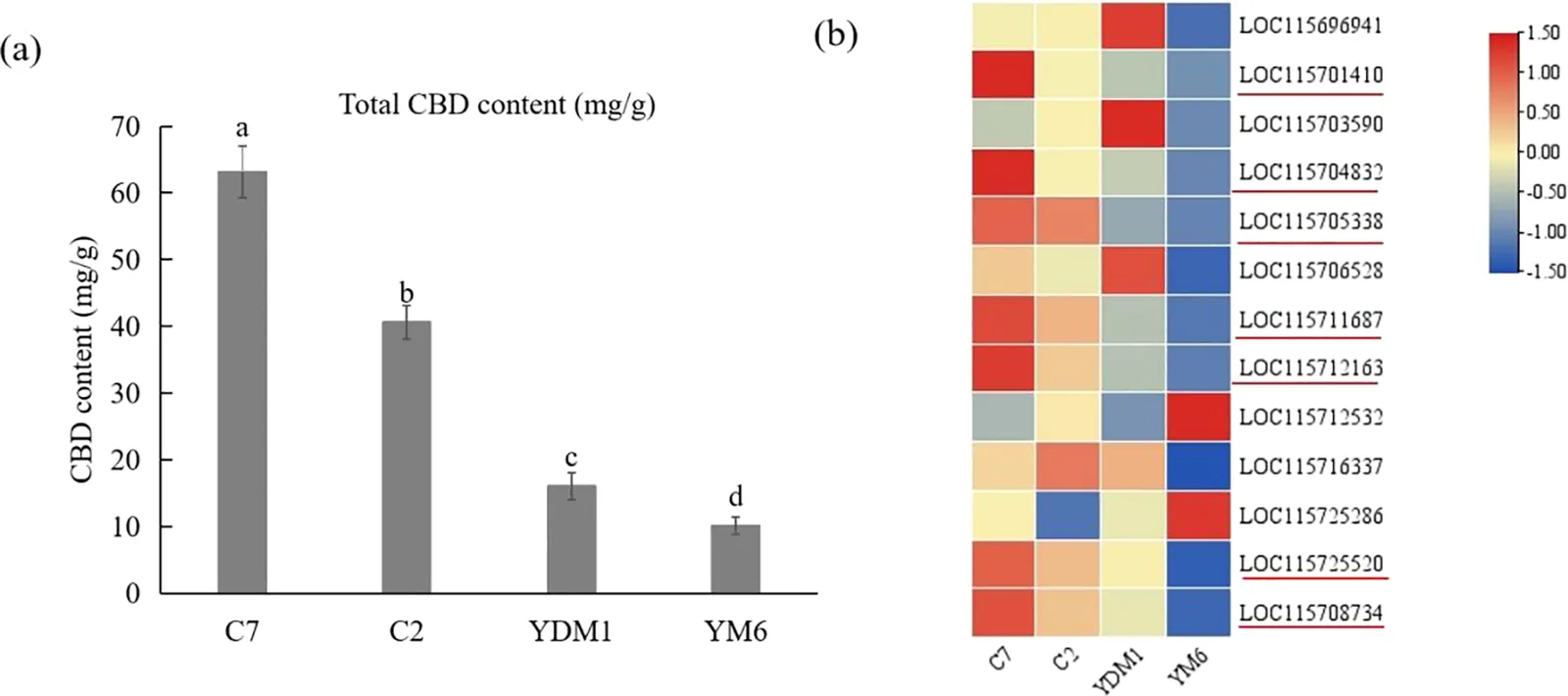
Correlation analysis between CBD accumulation and transcription factor expression in four cannabis varieties. **(a)** Measurement of CBD content in female flowers from four varieties. Data are means (± SD). Each experiment included three biological replicates. **(b)** Heatmap of expression levels of TF genes of female flower tissues in four varieties ‘C7’, ‘C2’, ‘YDM1’, and ‘YM6’ using qRT-PCR. The underlined genes indicated that their gene expression levels showed similar tendency to the CBD content in four varieties.

To further investigate the potential roles of these 13 TFs in the CBD biosynthesis pathway, the expression patterns of female flowers from the four varieties were analyzed. Significant differences in CBD contents were observed among the varieties ‘C7’, ‘C2, ‘ ‘YDM1, ‘ and ‘YM6’, with ‘C7’ having highest CBD content, followed by ‘C2’, ‘YDM1,’ and ‘YM6’ (**Figure 5a**). Among the 13 TFs, 7 upregulated genes had similar expression profiles and their expression levels in the female flower tissues of the four varieties showed a similar trend to CBD content, indicating that these 7 TFs may be the key putative regulators of CBD biosynthesis (**Figure 5b**).

### 3.5 Verification of TFs regulating CBDA biosynthesis

**Figure 6:**
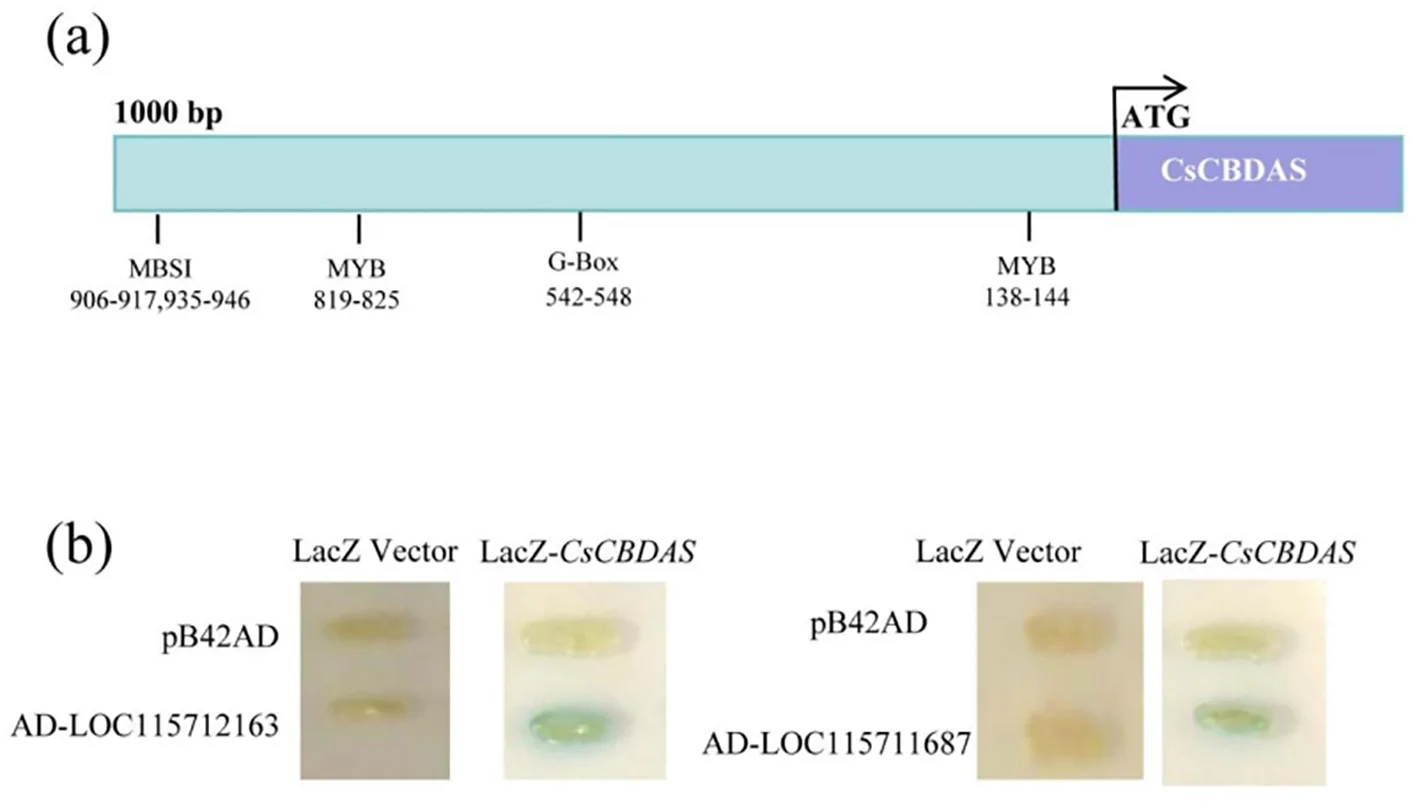
CsMYB6 (LOC115712163) and CsMYB29 (LOC115711687) bind to the promoter of *CsCBDAS*. **(a)** Schematic representation of cis-acting elements in the *CsCBDAS* promoter. Putative TF-binding sites, including MYB (138–144 and 819-825), MBSI (906–917 and 935-946), and G-Box (542-548) motifs, are indicated. **(b)** Y1H assay verifying the binding of CsMYB6 (AD-LOC115712163) and CsMYB29 (AD-LOC115711687) to the *CsCBDAS* promoter (LacZ-CsCBDAS). Empty vectors (pB42AD and LacZ Vector) were used as negative controls. Blue/green staining indicates positive protein-DNA interaction.

**Figure 7:**
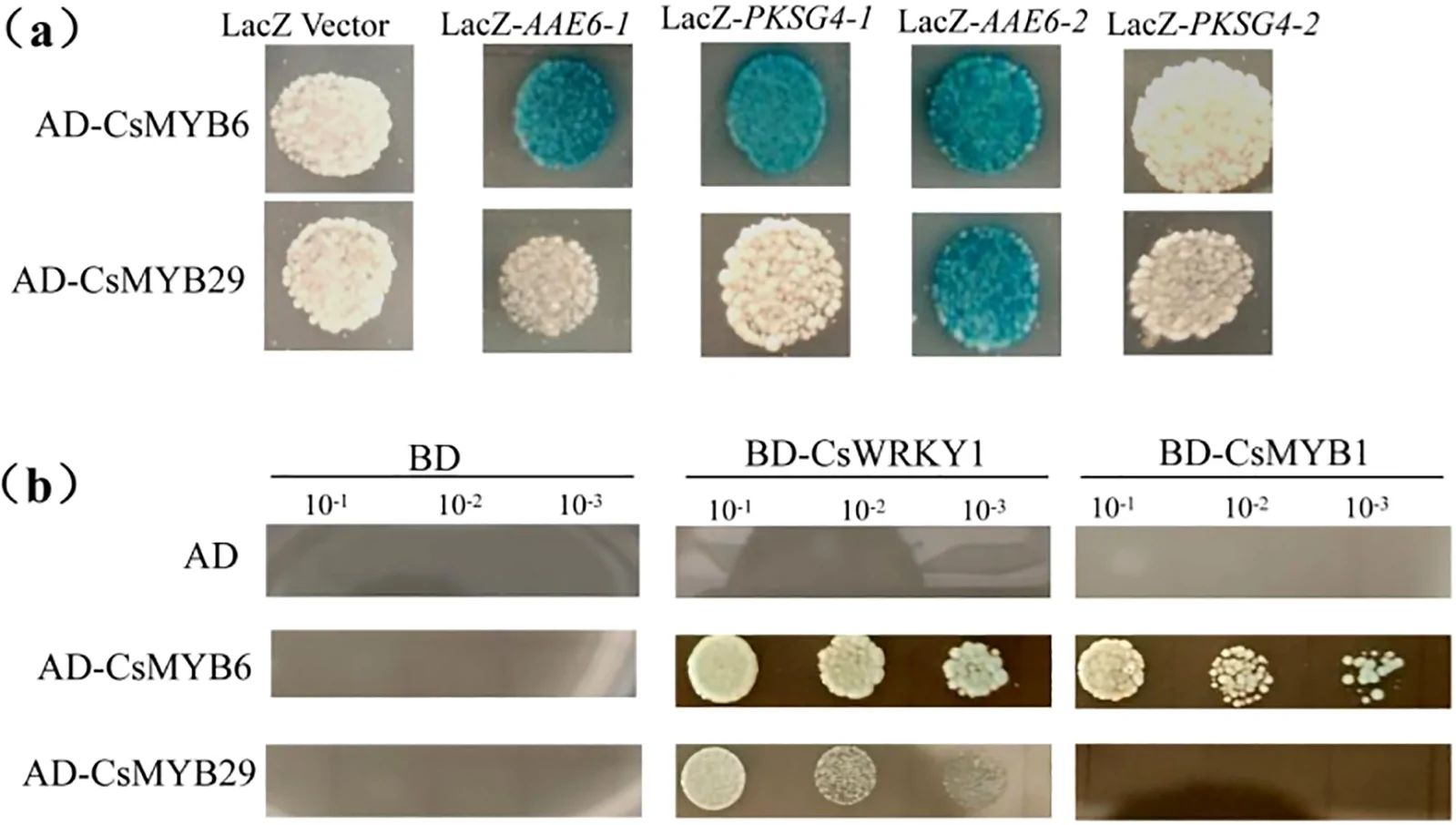
CsMYB6 and CsMYB29 interact with promoters of CBD biosynthetic genes and their regulatory proteins. **(a)** Yeast one-hybrid (Y1H) assays verifying the binding of CsMYB6 and CsMYB29 to four promoter fragments derived from CBD biosynthetic genes (*AAE6-1*, *PKSG4-1*, *AAE6-2*, *PKSG4-2*). Blue colonies indicate positive physical interactions between transcription factors and target promoter sequences. **(b)** Yeast two-hybrid (Y2H) assays confirming protein–protein interactions between CsMYB6/CsMYB29 and two CBD biosynthetic regulators (CsWRKY1 and CsMYB1). Serially diluted yeast suspensions (10^-1^, 10^-2^, 10^-3^) were spotted onto selective medium; empty BD vector controls exclude transcriptional autoactivation.

After analyzing the expression patterns and correlations between the transcript levels of the TFs and enzyme genes for CBD biosynthesis (**Figures 4**, **5**), we investigated whether the TFs directly activated the transcription of these enzyme genes. Given the important roles of *CsCBDAS* in the biosynthetic pathway of CBD (Onofri et al., 2015; Zirpel et al., 2018), we conducted a Y1H assay to investigate the relationship between seven TFs and *CsCBDAS*. Different TFs preferentially bind to different cis-elements, and the promoter of *CsCBDAS* was predicted to have cis-elements, including G-Box, MBSI, and MYB binding sites, using the software TBtool (Chen et al., 2020) (**Figure 6a**). The Y1H assay revealed that two TFs could bind to the promoter of *CsCBDAS*, whereas the other five TFs could not (**Figure 6**; **Supplementary Figure 6**), indicating that *CsMYB29* and *CsMYB6* may be involved in the CBD biosynthesis pathway by directly regulating the expression of *CsCBDAS*. To further investigate the role of the two TFs in CBD biosynthesis, we analyzed the relationship between these two TFs and the other four biosynthetic enzyme genes based on correlation analysis. The results showed that CsMYB6 can bind to the promoters of *CsAAE6-1*, *CsAAE6-2*, and *CsPKSG4–1* and that CsMYB29 only binds to the promoter of *CsAAE6-2* (**Figure 7a**).

CsWRKY1 and CsMYB1 have previously been identified as regulators that can bind to the promoter of *CsCBDAS* (Liu et al., 2021). Considering the complicated regulatory network involved in the biosynthesis of secondary metabolites in plants (Zheng et al., 2023), the relationship between the four TFs (CsWRKY1, CsMYB1, CsMYB6, and CsMYB29) was further investigated. Y2H assays showed that CsMYB6 could interact with CsWRKY1 and CsMYB1, whereas CsMYB29 could only interact with CsWRKY1 (**Figure 7b**).

### 3.6 Subcellular localization analysis of CsMYB29 and CsMYB6

**Figure 8:**
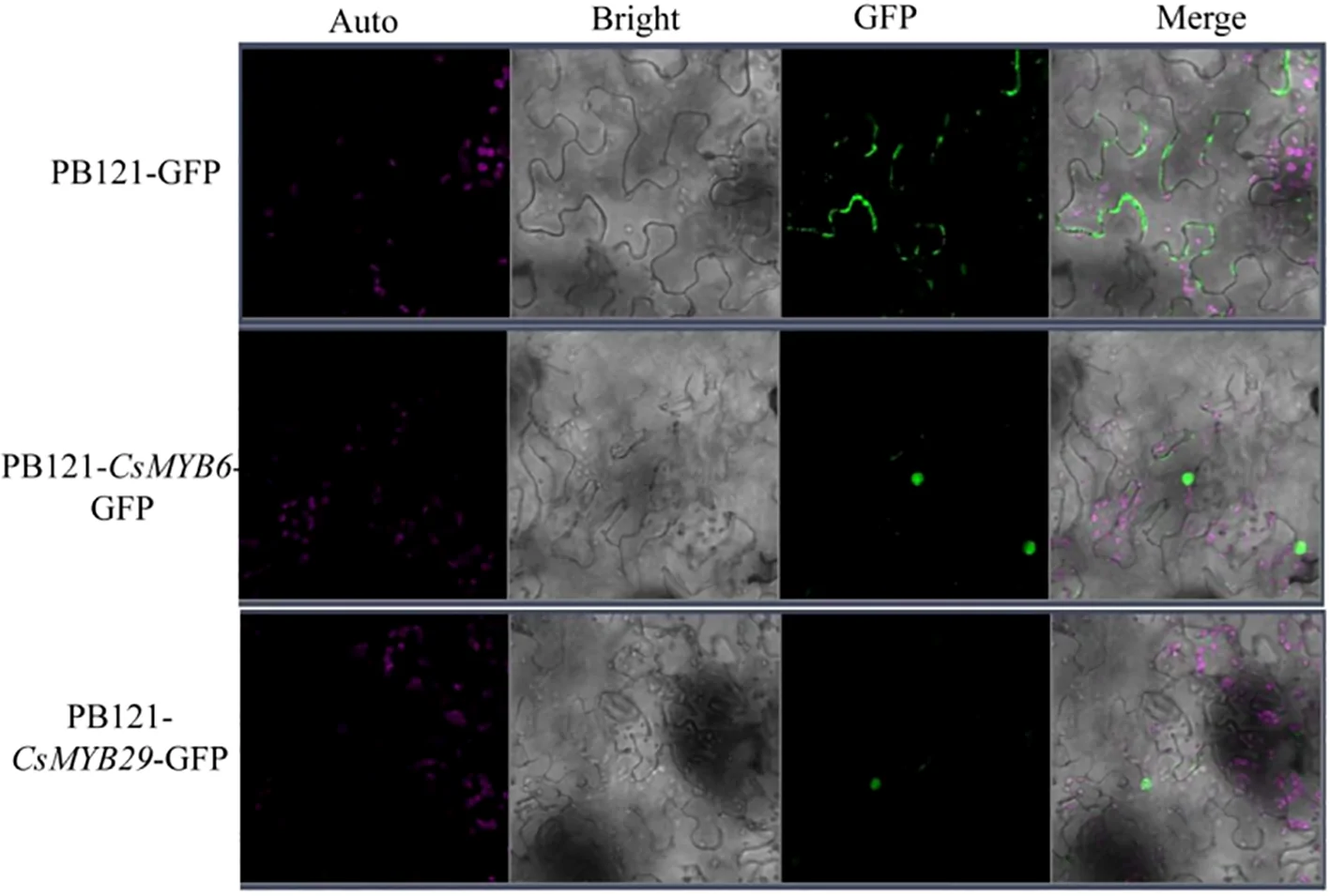
Subcellular localization analysis of CsMYB6 and CsMYB29 in tobacco leaves. The fusion constructs PB121-CsMYB6-GFP and PB121-CsMYB29-GFP, as well as the empty vector PB121-GFP (positive control), were transiently expressed in tobacco leaf epidermal cells. GFP fluorescence signals were observed by laser scanning confocal microscopy. Auto, chlorophyll autofluorescence; Bright, bright-field image; GFP, GFP fluorescence; Merge, overlay of multiple channels. Scale bars: 20 µ.

Typically, TFs are predicted to localize to the nuclei of plant cells. To determine the subcellular localization of these TFs, we analyzed the subcellular localization of CsMYB29 and CsMYB6 in tobacco leaves using the empty vector pBI121-GFP as a control. The results showed that CsMYB29 and CsMYB6 were localized in the nuclei of tobacco leaf cells (**Figure 8**). These results suggest that CsMYB6 and CsMYB29 function as potential regulators of CBD biosynthesis.

## 4 Discussion

Enhancing the CBD content of cannabis plants is a major breeding goal in China, where the CBD content of the main cultivated variety is approximately 1% (Zhang et al., 2019). Traditional breeding strategies have played an important role in the genetic improvement of CBD content in cannabis. However, the limitations of traditional breeding methods, such as lengthy breeding cycles, low accuracy, high randomness, and the need for continuous selective breeding, cannot be ignored (Guo et al., 2023). Compared with traditional breeding, molecular breeding enables precision variety improvement, for which mining key regulatory genes is an essential prerequisite. In this work, we combined STEM clustering, expression profiling, correlation analysis and yeast one-hybrid assays to systematically screen transcription factors (TFs) governing CBD biosynthesis. These candidate genes provide valuable genetic resources for breeding high-CBD cannabis varieties.

MYB TFs are well documented to modulate phenolic metabolism in medicinal plants via binding to target gene promoters (Thakur and Vasudev, 2022). Representative examples include SmMYB36, which activates tanshinone biosynthetic genes *SmDXS2*, *SmGGPPS1*, and *SmCPS1* (Ding et al., 2017), and SbMYB3, which upregulates flavonoid synthase gene *SbFNSII-2* and increases baicalin production (Fang et al., 2022). Here, two flower-preferential MYB genes, *CsMYB29* and *CsMYB6*, exhibited a consistent trend with total CBD content, whereas no consistent trend was observed with THC content (**Figures 3**, **4**; **Supplementary Figures 3**, **4**). Meanwhile, both proteins directly bind to the promoters of core CBD biosynthetic genes *CsCBDAS* and *CsAAE6-2* (Stout et al., 2012; Onofri et al., 2015; Zirpel et al., 2018) (**Figures 6**, **7a**). In addition, CsMYB29 and CsMYB6 physically interact with two previously characterized cannabinoid regulators CsMYB1 and CsWRKY1 (Liu et al., 2021) (**Figure 7b**). Collectively, our results suggest that CsMYB6 and CsMYB29 act as critical putative regulators of the CBD biosynthesis rather than functioning as general regulators of cannabinoid synthesis, laying a foundation for dissecting its transcriptional regulatory network. In addition, various specialized metabolites in cannabis, including CBD, are synthesized, stored, and secreted by glandular trichomes, whose density directly influences the CBD content (Livingston et al., 2020). Multiple MYB members are known to control trichome formation in Arabidopsis, cotton, and *Artemisia annua* L (Qin et al., 2021; Shangguan et al., 2021; Xu et al., 2023). Whether CsMYB6 and CsMYB29 participate in trichome development in cannabis awaits further transgenic validation.

Five additional candidate TFs displayed positive expression correlations with CBD levels and several biosynthetic structural genes, despite failing to interact with the promoter (**Figure 4**; **Supplementary Figure 3**). These TFs may modulate pathway genes through other promoter targets or regulate CBD biosynthesis independently of *CsCBDAS* expression, which also requires further investigation. Among them, *CsMYB106* (LOC115701410) was identified as a possible regulator of CBD biosynthesis through STEM, correlation, and expression profiling analyses (**Figures 2**–**4**). Consistent with earlier reports describing CsMYB106 as an putative regulator of CBD biosynthesis and glandular trichome development (Yin et al., 2022; Haiden et al., 2022), this gene represents a promising candidate for transgenic improvement of CBD content. Beyond these seven positively correlated genes, it is also worth noting that down-regulated genes, they may shape the regulatory network by repressing competing metabolic routes or mediating feedback inhibition, and their biological functions should be explored in follow-up research.

Notably, STEM analysis was originally developed for short time-series datasets. We adopted this tool for non-temporal expression data for two practical reasons: limited sample quantity precluded robust WGCNA, and previous work has proven STEM effective for identifying coordinated expression trends in non-temporal comparative transcriptomics (Zhao et al., 2019). Although we verified candidate interactions using Y1H and Y2H assays in the present study, transgenic functional characterization is still required to consolidate these findings.

## 5 Conclusion

**Figure 9:**
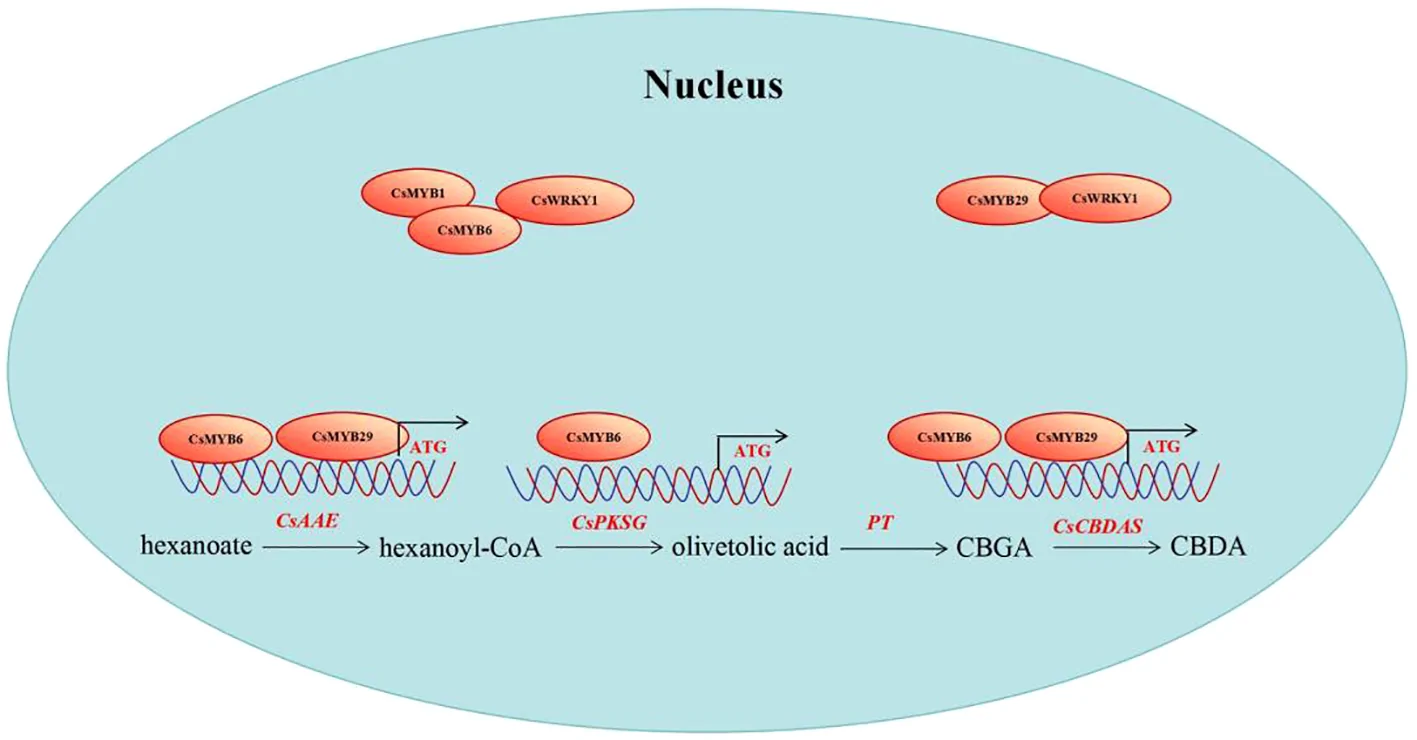
A proposed regulatory model illustrating the putative roles of CsMYB6 and CsMYB29 in CBD biosynthesis. As two transcription factors specifically expressed in female flowers, CsMYB6 and CsMYB29 exert dual regulatory functions. On the one hand, they directly bind to the promoter regions of CBD biosynthetic structural genes CsAAE, CsPKSG, and CsCBDAS to activate gene transcription. On the other hand, CsMYB6 and CsMYB29 physically interact with two known regulators of CBD biosynthesis, CsMYB1 and CsWRKY1, forming protein complexes to co-modulate the metabolic pathway for CBD production.

This study identified two putative TFs involved in CBD biosynthesis and revealed a complex transcriptional network underlying CBD biosynthesis. Seven TFs were identified as key putative regulators of CBD biosynthesis based on correlation analysis and qRT–PCR. Among these, we demonstrated that CsMYB6 interacts with both CsWRKY1 and CsMYB1, and binds to the promoters of *CsCBDAS*, *CsAAE6-1*, *CsAAE6-2*, and *CsPKSG4-1.* In parallel, CsMYB29 was found to interact with CsMYB1 and bind to the promoters of *CsCBDAS* and *CsAAE6-2* (**Figure 9**). Although additional research, such as verification of genetic modifications, is warranted to fully understand the underlying mechanisms by which these TFs regulate CBD biosynthesis, our study contributes to the existing body of knowledge on the regulatory mechanisms involved in the CBD biosynthetic pathway.

## Data Availability

The datasets presented in this study can be found in online repositories. The names of the repository/repositories and accession number(s) can be found below: https://www.ncbi.nlm.nih.gov/, PRJNA1173925.
