# Supplementary material for: Comparative transcriptome analysis reveals putative transcription factors components of the regulatory network for cannabidiol biosynthesis in Cannabis sativa L
Source: Front Plant Sci. 2026 Aug 31;17:1914974. doi: 10.3389/fpls.2026.1914974 (PMC13572606; doi:10.3389/fpls.2026.1914974)
Supplement: Supplementary file 1 [file DataSheet1.doc]

Table S1 The primers used in this study

| Primer | F (5'to3') | R (5'to3') | | Usage |
| --- | --- | --- | --- | --- |
| LOC115696941 | GTCGGTTACCGTCAATGTGG | AGTGAGGATGCTCGCTTGTA | qRT-PCR | |
| LOC115701410 | CTGCGACAAAGTGGGATTGA | GGTAAAGCTCGCCAACTTCC |  | |
| LOC115703590 | CCACCTCACAATGGCTCCTA | GTGGTGTCCCGCCTTTATTC |  | |
| LOC115704832 | ACTGTGCTTGGCATTGAAGG | TAGAGACACGCAGTGAAGGG |  | |
| LOC115705338 | CTGGGTCTGGGTCTGAGTC | CTCCTCCTCATCAAGTCCCA |  | |
| LOC115706528 | TGGAAGAGAATTGCGGTGAT | CCAGTGGTGCCAATCCTTTC |  | |
| LOC115711687 | GAGAGTTCAGCCCTGAGGAA | TGTTCCTCGTTTCTTCAACCG |  | |
| LOC115712163 | TGGTCCATAGAAGAGGACCAG | TTTCCACATCGAAGCAACCC |  | |
| LOC115712532 | AGTCACCCAACAAAGCCAAC | TGTTCCGTTCCTTCTCCGAA |  | |
| LOC115716337 | ATCCGGACTCCATACAACCC | GCATGAGGTCATTCCCAACC |  | |
| LOC115725286 | GATAAAGAGAGGGCCGTGGA | TAGTTCATCCACCGGAGACG |  | |
| LOC115725520 | TGGGAGTGAGATGATGAATAAACG | TTCTTCGTCGCAGAGGCAAA |  | |
| LOC115708734 | CGAAGTGGTAGTAAGTGACTTGG | ACTCAAGAGTTGCAGCATCA |  | |
| EF1a | CAGTTGAGATGCACCACGAG | TTGCCAATTTGACCAGGGTG |  | |
| LOC115711687-SL | gagaacacgggggactctagaATGGGTAGGACTCCATGCTGTG | gctcaccatggatcctctagaCATATATCTTAAGACATCTTCCAAACAATC | Subcellular localization | |
| LOC115712163-SL | gagaacacgggggactctagaATGAGAAAACCTTGTTCTGAAAAGC | gctcaccatggatcctctagaTATGAAGAGAGGGAGTGTTGGAGC |  | |
| LOC115711687-Y1H | TGCCTCTCCCGAATTCATGGGTAGGACTCCATGCTGTGACCAAAAAG | TCCAAAGCTTCTCGAGGTTTGGAAGATGTCTTAAGATATATG | Yeast one-hybrid | |
| LOC115712163-Y1H | TGCCTCTCCCGAATTCATGAGAAAACCTTGTTGTGAAAAGCAAGAC | TCCAAAGCTTCTCGAGCAGCTCCAACACTCCCTCTCTTCATA | Yeast one-hybrid | |
| LOC115701410-Y1H | TGCCTCTCCCGAATTCATGATTCAACCAAAGAAATTCAG | TCCAAAGCTTCTCGAGTTATATATTAAGGAGCTCCTC | Yeast one-hybrid | |
| LOC115708734-Y1H | TGCCTCTCCCGAATTCATGGGAAGGACACCCTGCTGCAACGTC | TCCAAAGCTTCTCGAGCTCAAGAGTTGCAGCATCAAAGAATTC | Yeast one-hybrid | |
| LOC115704832-Y1H | TGCCTCTCCCGAATTCATGGAGCAACCTCTATTCGCC | TCCAAAGCTTCTCGAGCTAGAGACACGCAGTGAAGG | Yeast one-hybrid | |
| LOC115705338-Y1H | TGCCTCTCCCGAATTCATGATTCAACCAAAGAAATTCAG | TCCAAAGCTTCTCGAGTTATATATTAAGGAGCTCCTC | Yeast one-hybrid | |
| LOC115725520-Y1H | TGCCTCTCCCGAATTCATGATGAATAAACGAATTCGTC | TCCAAAGCTTCTCGAGTCATTGGTGGCTTAATTCCACTAG | Yeast one-hybrid | |
| CsCBDAS-Y1H | ATCTGTCGACCTCGAGTAGGCGTTGAGATACAATGTTTTC | GAGCACATGCCTCGAGTTTTCTTTAGTTCTAATAATTTTTG | Yeast one-hybrid | |
| LOC115711687-AD | GGAGGCCAGTGAATTCATGGGTAGGACTCCATGCTGTG | CGAGCTCGATGGATCCTTACATATATCTTAAGACATCTTCCAAACAATC | Yeast two-hybrid | |
| LOC115712163-AD | GGAGGCCAGTGAATTCATGAGAAAACCTTGTTCTGAAAAGC | CGAGCTCGATGGATCCTTATATGAAGAGAGGGAGTGTTGGAGC | Yeast two-hybrid | |
| CsMYB1-BD | CATGGAGGCCGAATTCATGGATAGGATCAAAGGTCCATGG | GCAGGTCGACGGATCCCTACTCGATCTTGCCAAGTCCC | Yeast two-hybrid | |
| CsWRKY1-BD | CATGGAGGCCGAATTCATGGAAGCTGCCGCTGCT | GCAGGTCGACGGATCCTTAAGGACCTGTTAAGATTCTAC | Yeast two-hybrid | |

Table S2 Results of transcriptome data of different samples

| Sample | raw_reads | raw_bases | clean_reads | clean_bases | valid_bases | Q30 | Map rate | GC |
| --- | --- | --- | --- | --- | --- | --- | --- | --- |
| C4_1F | 57.13M | 8.57G | 55.29M | 8.13G | 94.85% | 95.23% | 92.88% | 43.97% |
| C4_1L | 49.06M | 7.36G | 46.71M | 6.83G | 92.88% | 93.67% | 92.45% | 44.19% |
| C4_1R | 54.89M | 8.23G | 52.94M | 7.76G | 94.31% | 94.61% | 91.33% | 43.30% |
| C4_1S | 49.64M | 7.45G | 48.19M | 7.08G | 95.06% | 95.45% | 92.49% | 43.52% |
| C4_2F | 51.25M | 7.69G | 49.36M | 7.25G | 94.26% | 94.60% | 92.91% | 43.63% |
| C4_2L | 54.93M | 8.24G | 53.09M | 7.79G | 94.59% | 94.86% | 92.95% | 44.37% |
| C4_2R | 43.51M | 6.53G | 42.02M | 6.16G | 94.45% | 94.97% | 89.85% | 43.58% |
| C4_2S | 57.17M | 8.58G | 55.37M | 8.15G | 94.99% | 95.28% | 92.68% | 43.62% |
| C4_3F | 51.71M | 7.76G | 49.91M | 7.32G | 94.34% | 94.71% | 92.71% | 43.65% |
| C4_3L | 52.60M | 7.89G | 50.16M | 7.36G | 93.25% | 93.79% | 92.9% | 44.38% |
| C4_3R | 48.99M | 7.35G | 47.51M | 6.96G | 94.64% | 95.01% | 91.05% | 43.47% |
| C4_3S | 52.47M | 7.87G | 50.75M | 7.46G | 94.76% | 95.13% | 92.57% | 43.65% |
| Mean | 51.94M | 7.79G | 50.11M | 7.35G | 94.36% | 94.77% | 92.24% | 43.77% |

**Fig. S1.** A schematic diagram of the cannabidiols biosynthetic pathway

**Fig. S2.** The total THC content of four tissues in C4 female plants

Different letters indicate significant difference between different tissues (P<0.05). Data are means (±SD), and each experiment included three biological replicates.

**Fig. S3.** The THC content of female flower in four cannabis cultivar ‘C7’, ‘C2’,‘YDM1’ and ‘YM6’

Different letters indicate significant difference between different tissues (P<0.05). Data are means (±SD), and each experiment included three biological replicates.

**Fig. S4.** The FPKM values of CsMYB6 and CsMYB29 of four tissues in C4 female plants

Different letters indicate significant difference between different tissues (P<0.05). Data are means (±SD), and each experiment included three biological replicates.

**Fig. S5.** Gene Regulatory Network between transcription factor and CBD biosynthetic enzyme genes of Profile 8

Omics 1: enzyme gene for CBD biosynthesis; Omics 2: transcription factor

**Fig. S6.** Verification of the interaction relationship of TFs and *CsCBDAS*
